# A mathematical model of marine bacteriophage evolution

**DOI:** 10.1098/rsos.171661

**Published:** 2018-03-07

**Authors:** Silvia Pagliarini, Andrei Korobeinikov

**Affiliations:** 1 Department of Computer Science, Univestità degli Studi di Verona, Verona, Italy; 2 Centre de Recerca Matemàtica, Campus de Bellaterra, 08193 Barcelona, Spain; 3 Departament de Matemàtiques, Universitat Autònoma de Barcelona, 08193 Barcelona, Spain

**Keywords:** viral evolution, phenotype space, travelling wave, Darwinian fitness, paradox of enrichment

## Abstract

To explore how particularities of a host cell–virus system, and in particular host cell replication, affect viral evolution, in this paper we formulate a mathematical model of marine bacteriophage evolution. The intrinsic simplicity of real-life phage–bacteria systems, and in particular aquatic systems, for which the assumption of homogeneous mixing is well justified, allows for a reasonably simple model. The model constructed in this paper is based upon the Beretta–Kuang model of bacteria–phage interaction in an aquatic environment (Beretta & Kuang 1998 *Math. Biosci.* **149**, 57–76. (doi:10.1016/S0025-5564(97)10015-3)). Compared to the original Beretta–Kuang model, the model assumes the existence of a multitude of viral variants which correspond to continuously distributed phenotypes. It is noteworthy that the model is mechanistic (at least as far as the Beretta–Kuang model is mechanistic). Moreover, this model does not include any explicit law or mechanism of evolution; instead it is assumed, in agreement with the principles of Darwinian evolution, that evolution in this system can occur as a result of random mutations and natural selection. Simulations with a simplistic linear fitness landscape (which is chosen for the convenience of demonstration only and is not related to any real-life system) show that a pulse-type travelling wave moving towards increasing Darwinian fitness appears in the phenotype space. This implies that the overall fitness of a viral quasi-species steadily increases with time. That is, the simulations demonstrate that for an uneven fitness landscape random mutations combined with a mechanism of natural selection (for this particular system this is given by the conspecific competition for the resource) lead to the Darwinian evolution. It is noteworthy that in this system the speed of propagation of this wave (and hence the rate of evolution) is not constant but varies, depending on the current viral fitness and the abundance of susceptible bacteria. A specific feature of the original Beretta–Kuang model is that this model exhibits a supercritical Hopf bifurcation, leading to the loss of stability and the rise of self-sustained oscillations in the system. This phenomenon corresponds to the paradox of enrichment in the system. It is remarkable that under the conditions that ensure the bifurcation in the Beretta-Kuang model, the viral evolution model formulated in this paper also exhibits a rise in self-sustained oscillations of the abundance of all interacting populations. The propagation of the travelling wave, however, remains stable under these conditions. The only visible impact of the oscillations on viral evolution is a lower speed of the evolution.

## 1. Introduction

Owing to very high replication rate combined with high mutability, viruses are able to evolve considerably faster than larger cellular organisms. In many instances, viral mutations and evolution are responsible for emergence of new pathogens and determine pathogenesis of existing diseases. The high level of evolvability also makes viruses a good model in evolutionary biology. Moreover, study of viral mutations and evolution is also indispensable for designing and estimating efficacy of antiviral therapies and vaccination strategies, for determining the risk of the development of drug resistance, and other similar tasks.

Accordingly, during the last 20 years, a number of mathematical models of viral evolution have been suggested. Probably the first step in this direction was done by Pease [1], who formulated a simplistic model of inter-host viral evolution to study evolutionary changes in influenza A virus. Later, Tsimring *et al.* [2] suggested an elegant model of within-host RNA virus evolution where virus types were assumed to be distributed in a one-dimensional continuous space according to their fitness. The use of a continuous fitness space allows representation of random mutations by diffusion operator. The Tsimring–Levine–Kessler model (the TLK model) exhibited a solution in the form of a pulse-type travelling wave of evolution moving in the space towards higher fitness. Sasaki and his collaborators [3–5] used an intra-host pathogen–antibodies model with a discrete or continuous one-dimensional strain space to study antigenic drift and host–virus co-evolution. These models exhibit evolution of antigen variants in the form of a pulse-type travelling wave in the strain space. Lin *et al.* [6] studied inter-host antigenic drift, using a generalization of the classical SIR model where mutations were included as a diffusion process in a one-dimensional continuous phenotype space. This model also exhibits travelling wave solutions.

While these and other recently suggested models brought important insights into the problem, nearly all these models also possess an apparent shortcoming. Specifically, the above-mentioned models, as well as the majority of other recently proposed models of viral evolution, are essentially phenomenological. That is, while such models can produce qualitative description of a process, biological interpretation of obtained results, as well as model parameters, is dubious.

In contrast to phenomenological models, the so-called mechanistic models are based upon a clearly defined set of assumptions and hypotheses (the ‘first principles’). For mechanistic models, physical or biological interpretation of results and model parameters is usually straightforward. This makes mechanistic models suitable for complementing real-life experiments and verifying hypotheses, as well as for quantitative (rather than qualitative) studies. However, since mechanistic models necessarily take into consideration particularities of studied physical or biological systems, they are usually considerably more complex than phenomenological models.

A reasonably simple mechanistic model of HIV within-host evolution was recently suggested by Korobeinikov & Dempsey [7]. This model is based upon the Nowak and May model of HIV intra-host dynamics [8] and is mechanistic (as far as the Nowak–May model is mechanistic). Several variants of this model were considered [9–12]. However, all these variants are also based on the Nowak–May model, and hence their applicability to other virus–host systems is questionable.

Compared to other viral dynamics models, the most notable particularity of the Nowak–May model is the target cell reproduction mode. This model is specifically designed for HIV infection and postulates that the target cells (which in this case are T-helper lymphocytes) do not reproduce and that, instead, there is a continuous influx of the cells into the system (from the thymus, where they mature). In contrast, in the majority of virus–host systems the hosts reproduce within the system, whereas their immigration into the system can be assumed complementary.

To explore potential impacts of particularities of a virus–host system, and in particular impacts of the host reproduction modes, on viral evolution, in this paper we construct a model of marine bacteriophage evolution that is based upon a model of marine bacteriophage dynamics suggested by Beretta & Kuang [13]. The major difference between the Beretta–Kuang model and the Nowak–May model is the host cell reproduction mode. While this difference may appear insignificant, it results in a distinctive dynamics exhibited by the Beretta–Kuang model that makes it particularly attractive for our objectives. Specifically, the Beretta–Kuang model exhibits the effect of enrichment, that is the loss of stability and a rise of self-sustained oscillations in response to an increment of the environment carrying capacity.

A comparative simplicity of the aquatic bacteria–phages systems makes them particularly suitable for our objectives. Thus, real-life marine bacteria–phages systems typically comprise bacteria, phages and nutrients. These systems do not include complications such as immune response, which is an essential component of real-life virus–human and virus–animal interactions. Furthermore, phages are usually host-specific and form stable phage–host bacteria couples. Moreover, aquatic environments are fairly homogeneous (at least on a limited spacial scale that, however, is sufficiently large for modelling bacteria–phages interaction), and the assumption of homogeneous distribution of all components is well justified.

### 1.1. Background on bacteriophages

Bacteriophages are small viruses that infect and replicate within bacteria, effectively killing their bacterial host. Bacteriophages were discovered independently by Frederick Twort in 1915 and by Félix d’Herelle in 1917. However, the viral nature of phages was recognized only after invention of the electron microscope.

Bacteriophages are one of the most widespread and diverse entities in the biosphere [14]. Sea water is one of the densest natural sources for phages and other viruses, where up to 9×10^8^ virions per millilitre have been found in microbial mats at the surface [15]. Moreover, it was estimated that up to 70% of marine bacteria may be infected by phages [16].

Scientific interest in phages was significantly promoted by their potential use against bacterial infectious diseases. Probably the first advance in this direction was made by d’Herelle, who used phages to treat dysentery. However, d’Herelle did not published this study immediately, and the first published report about the therapeutic use of bacteriophages was made by Richard Bruynoghe and Joseph Maisin in 1921, who used phages to treat a staphylococcal skin disease [17]. Research in this direction ceased in the 1960s when antibiotics had become widely available. However, low costs of such treatment, as well as the development of antibiotic resistance by bacteria, revitalized research in this direction.

A typical phage consists of a protein hull (or capsid) and the enclosed genetic material. For the majority of the known phages the genetic material consists of double-stranded DNA, but phages with single-stranded DNA and RNA genomes are also known. Some phages also have a ‘tail’ that serves for injecting the genetic material into a bacterium. By their mode of reproduction, phages can be roughly divided into lytic and non-lytic (or temperate) types. The lytic phages are a highly virulent type that start reproducing immediately after infecting a bacterium and after a short time lyse (destroy) the host bacterium, releasing new free phages at the instance of bacterium death. The non-lytic or temperate phages either integrate their genetic material into the chromosomal DNA of the host or establish themselves as plasmids, and then are copied with every cell division together with the DNA of the host cell. When the host cell starts to show signs of stress (meaning it might be about to die), the endogenous phages become active again and start their reproductive cycle, resulting in the lysis of the host cell.

In this paper, we consider phages with the lytic reproduction cycle only.

## 2. Model

We use the Beretta–Kuang model of lytic marine bacteriophagesdynamics [13] as a basis for constructing a model of bacteriophage evolution. The Beretta–Kuang model describes the dynamics of three interacting populations, namely a population of susceptible target bacteria, of size (or concentration) *S*(*t*), a population of bacteria infected by the bacteriophages, of size (or concentration) *I*(*t*), and free bacteriophages of size (or concentration) *P*(*t*). All three populations are assumed to be homogeneous. The model postulates that the susceptible bacteria replicate according to the logistic law, with *per capita* reproduction rate *α* and carrying capacity of the environment *C*. The susceptible bacteria are infected by free bacteriophages at bilinear rate *kSP*. Infected bacteria do not reproduce and die with *per capita* rate *λ*, releasing at the instance of death on average *b* phages. Free viruses die at *per capita* rate *μ*. Moreover, the model also takes into account that the free phage population decreases by those phages that infect bacteria. Accordingly, the model equations are

2.1 $$\begin{array}{rl} \frac{dS}{dt} & =\alpha S\left(1-\frac{S+I}{C}\right)-kSP,\\ \frac{dI}{dt} & =kSP-\lambda I,\\ \;\text{and}\;\frac{dP}{dt} & =b\lambda I-kSP-\mu P. \end{array}\}$$

An important feature of this model is that the model is based upon a clearly identified set of assumptions and hypotheses (the first principles); that is, model (2.1) is mechanistic.

The phase space of the model is $R_{\geq 0}^{3}$. The model always has an equilibrium state *E*_0_=(0,0,0) at the origin and a phage-free equilibrium state *E*_*f*_=(*C*,0,0). Apart from these two equilibrium states, the model can have a positive equilibrium state *E*_+_ with coordinates

$$S_{+}=\frac{C}{R_{0}},\;I_{+}=\frac{\alpha C}{\lambda R_{0}+\alpha }\;\frac{R_{0}-1}{R_{0}},\;P_{+}=\frac{\lambda R_{0}}{kC}I_{+},$$

where *R*_0_=(*b*−1)*kC*/*μ* is the phage’s basic reproduction number.

The model dynamics is determined by the basic reproduction number *R*_0_. In particular, if 0<*R*_0_≤1, then the phage-free equilibrium state *E*_*f*_ is globally asymptotically stable, and no positive equilibria exist. At *R*_0_=1, equilibrium states *E*_*f*_ and *E*_+_ merge, and a saddle–node bifurcation occurs. Positive equilibrium state *E*_+_ exists for all *R*_0_>1; *E*_*f*_ is unstable (a saddle point) for these *R*_0_.

As the basic reproduction number grows further, a supercritical Hopf bifurcation occurs in the system: equilibrium state *E*_+_ loses its stability, and a stable limit cycle appears in the phase space [13,18]. This phenomenon is usually referred to as the *paradox of enrichment* in ecology [19]. The possibility of the supercritical Hopf bifurcation in the Beretta–Kuang model is the principal difference between this model and simpler models, such as the Nowak–May model of HIV-1 dynamics that served as a basis for a model of viral evolution [7,9–11].

Model (2.1) postulates that all elements in each of the three populations are identical. In order to develop a model of bacteriophage evolution, let us assume, instead, that a multitude of viral genotypes exists and that several genotypes are simultaneously present in the environment. Each genotype is characterized by a set of phenotypic traits, which in this model framework is represented by parameters *k*, *λ*, *μ* and *b*.

It is reasonable to assume that the phenotypic traits of closely related genotypes are also close. To define distance between genotypes *A* and *B*, one can assume that the distance is inversely proportional to the probability of mutation of genotype *A* to *B* (and vice versa) [20]. Please note that such a definition implies that the probability of mutations A→B and B→A are equal. However, the equality does not necessary hold in every case. Nevertheless, if the probability of mutations A→B and B→A are not equal, then probabilities of mutations in a predefined direction can be used to define the distance. Then to take into account the disparity in the probabilities of mutations in the opposite direction one can use an asymmetric mutation operator.

The set of viral genotypes is discrete. However, due to phenotypical plasticity and stochasticity of the environment, values of parameters *k*,*λ*,*μ* and *b* corresponding to a specific viral genotype vary within certain tolerance intervals. Moreover, for closely related genotypes these intervals are likely to overlap. Therefore, we can use a continuous phenotype (or variant) space instead of a discrete genotype space. In the Beretta–Kuang model, a viral type is described by four parameters, *k*,*λ*,*μ* and *b*, and hence a continuous phenotype space should be of dimension up to 4.

A particular choice for a phenotype space, *Ω*, is arbitrary and to a large extent is a matter of convenience. As the simplest (and probably the most natural) choice, a four-dimensional real space $R^{4}={\left(-\infty ,+\infty \right)}^{4}$ can be used as the phenotype space. In this paper, for convenience, we assume that phenotype space is the positive four-dimensional orthant of the four-dimensional real space, $\Omega =R_{+}^{4}={\left(0,+\infty \right)}^{4}$, and *r*∈*Ω* is the space coordinate.

For distributed viral variants, we define in the variant (phenotype) space density distribution *p*(*r*,*t*), such that $P(t)=\int_{\Omega }p(r,t)\;dr$ holds. We also have to segregate the infected bacteria with respect to a viral variant that these are infected with, defining density distribution of the infected bacterial population *i*(*r*,*t*). (Hence, $I(t)=\int_{\Omega }i(r,t)\;dr$.) Thus, we have a model with three variables: the susceptible population *S*(*t*) (which is independent of viral phenotypes), the density distribution of infected population *i*(*r*,*t*) and the density distribution of free phages population *p*(*r*,*t*).

In order to model random mutations, let us assume that with a certain probability an infected bacterium can switch from producing the genotype that it is infected with to a different genotype. We assume that the probability of such a mutation is small and is quickly (e.g. exponentially) decreasing with the growth of the distance between the original and mutant genotypes. In the continuous phenotype space such random mutations can be modelled by an integral operator or by the dispersion (diffusion) operator [2–5,7]. An integral operator provides more flexibility and potentially can describe more complicated features of real-life systems (such as asymmetric mutations). However, the dispersion leads to a simple model, is easier for analysis and is ‘cheaper’ for numerical simulations. For simplicity, in this paper we prefer to use the dispersion operator.

Under these assumptions, the continuous phenotype space model of bacteriophage evolution is represented by the following system of integro-partial differential equations:

2.2 $$\begin{array}{rl} \frac{dS(t)}{dt} & =\alpha S(t)\left(1-\frac{1}{C}\left(S(t)+\int_{\Omega }i(r,t)\;dr\right)\right)-\int_{\Omega }k(r)p(r,t)S(t)\;dr,\\ \frac{\partial i(r,t)}{\partial t} & =k(r)p(r,t)S(t)-\lambda (r)i(r,t)+q\Delta i(r,t),\\ \;\text{and}\;\frac{\partial p(r,t)}{\partial t} & =-k(r)p(r,t)S(t)-\mu (r)p(r,t)+b(r)\lambda (r)i(r,t). \end{array}\}$$

Here the coefficient of dispersion *q* is proportional to the probability of mutation and is assumed to be variant-independent (constant) and small.

The model should be completed by initial and boundary conditions. For an unbounded phenotype space, it is natural to assume that i(∞,t)=p(∞,t)=0 holds everywhere at infinity. For a bounded phenotype space, Robin-type conditions,

$$q\frac{\partial i(r,t)}{\partial n}=i(r,t),\;q\frac{\partial p(r,t)}{\partial n}=p(r,t),$$

where ∂*i*(*r*,*t*)/∂*n*,∂*p*(*r*,*t*)/∂*n* are the normal derivatives to the boundary, can be applied.

Furthermore, functions *r*(*r*),*λ*(*r*),*μ*(*r*) and *b*(*r*) (that is, the *fitness landscape*) should be defined.

We have to stress that model (2.2) does not include any explicit law of evolution, or a mechanism of evolution. Instead it includes only a possibility of random mutations and a mechanism of natural selection (a possibility for conspecific competition for a limited resource). We assume that, in agreement with the Darwinian principles, these are sufficient to produce viral evolution.

It is also noteworthy that model (2.2) includes at least four time scales, namely life-spans of the susceptible bacteria, the infected bacteria and free phages and the characteristic time of evolution. This fact makes analysis of the model rather challenging.

## 3. Results

We mentioned above that in the model (2.2) framework each viral variant is described by four parameters, namely *k*,*λ*,*μ* and *b*. For the sake of simplicity, let us assume that only one of these four parameters, say *k*=*k*(*r*), is variable in phenotype space *Ω*, whereas *λ*,*μ* and *b* are variant-independent and constants. This assumption allows to reduce the dimension of phenotype space to one. Moreover, for simplicity let us assume that *k*=*ξr*. Then the variant-specific reproduction number *R*_0_(*r*), which can serve as a measure of the Darwinian fitness, is

$$R_{0}(r)=\frac{\left(b(r)-1)Ck(r\right)}{\mu (r)}=\frac{(b-1)C}{\mu }\xi r.$$

That is, the Darwinian fitness linearly grows in the phenotype space *Ω*. In numerical simulations we use *ξ*=0.002 ml (cells day)^−1^. The other parameters are as follows: *λ*=3 days^−1^, *μ*=20 days^−1^, *b*=149.254, *α*=1.5 days^−1^ and *C*=100 cells ml^−1^. (Please note that variable *r* and parameter *b* are non-dimensional.) For these values *R*_0_≈1.5 at *r*=1. In simulations the coefficient of diffusion *q*, which is proportional to the rate of mutation, is *q*=10^−6^ days^−1^.

In the simulations we assume that initially all bacteria are susceptible. That is, *S*(0)=*C*, whereas the initial distribution of infected bacteria is identically equal to zero. The initial distribution of free virus differs from zero only in a narrow vicinity of *r*=1. For the simulations, we use a finite interval *r*∈(0,*r*_*end*_) of phenotype space *Ω*, rather than the entire semi-axis (0,+∞), applying the Robin boundary conditions

$$q\frac{\partial i}{\partial r}=i{|}_{r=0},\;q\frac{\partial p}{\partial r}=p{|}_{r=0},\;q\frac{\partial i}{\partial r}=i{|}_{r=r_{\mathrm{end}}},\;q\frac{\partial p}{\partial r}=p{|}_{r=r_{\mathrm{end}}}$$

at both ends.

**Figure 1.**
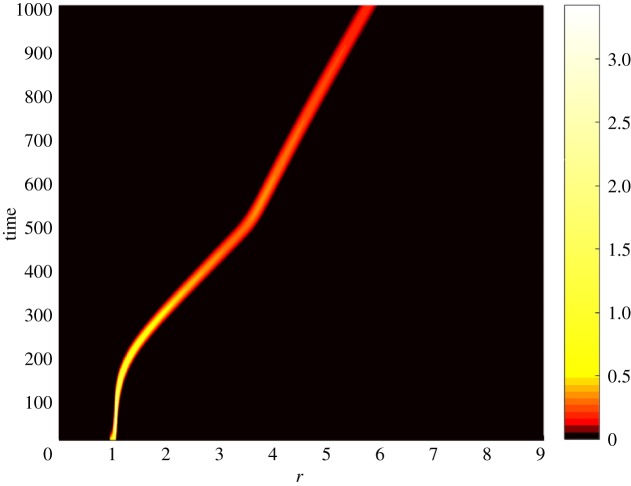
Density distribution of the infected population *i*(*r*,*t*) by viral variant in time. Here *q*=10^−6^ days^−1^, *α*=1.5 days^−1^, *C*= 100 cells ml^−1^, *λ*=3 days^−1^, *μ*=20 days^−1^, *b*=149.254 and *ξ*=0.002 ml cell day^−1^. The colours correspond to the density magnitude; see the legend on the right-hand side. Please note the formation of a pulse-type travelling wave moving in the phenotype space in the right-hand direction. The speed of the wave varies in time depending on the fitness of the virus and size of the infected population.

**Figure 2.**
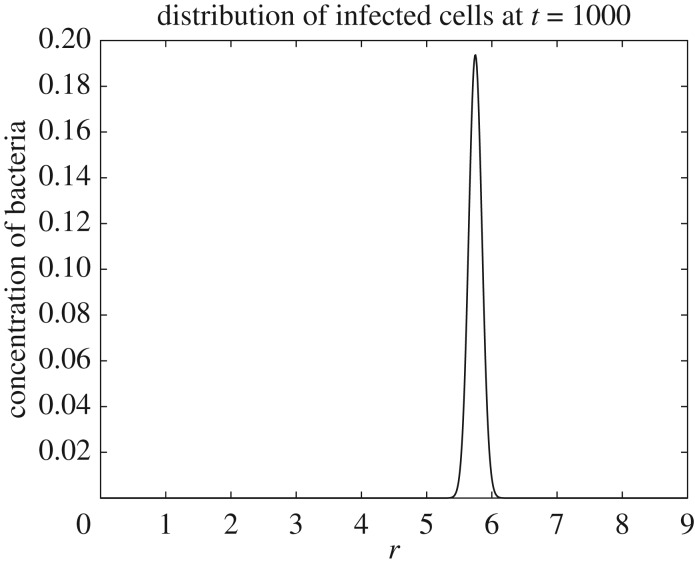
Distribution of the infected bacteria *i*(*r*,*t*) by viral variants they are infected with at *t*=1000 days.

Figure 1 shows distribution of infected bacteria in the viral variant space and time for the parameters above. In this figure, colours correspond to the density *i*(*r*,*t*) at a particular point. Thus, black colour corresponds to the zero level; see legend at the right-hand side of the panel for detail. The formation of a pulse-type travelling wave moving in the variant space towards higher Darwinian fitness (towards higher *R*_0_(*r*)) is clearly seen in this figure. This result indicates that for this particular fitness landscape the bacteriophages’ Darwinian fitness (which is represented by the basic reproduction number *R*_0_) is increasing in time. That is, the simulations confirm that, for an uneven fitness landscape, random mutations combined with natural selection results in evolution towards increasing Darwinian fitness. Figure 2 shows a typical profile of the travelling wave, that is a typical distribution of infected bacteria *i*(*r*,*t*) by viral variants they are infected with, at a particular time moment (figure 2 depicts the data for *t*=1000 days).

**Figure 3.**
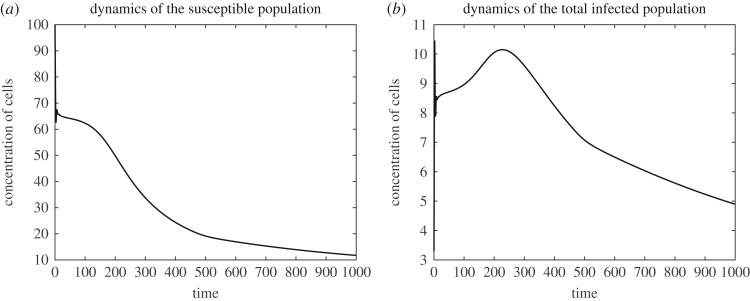
Dynamics of the susceptible population *S*(*t*) (*a*) and the total infected population $I(t)=\int_{\Omega }i(r,t)\;dr$ (*b*).

Figure 3*a*,*b* shows the dynamics of the susceptible population *S*(*t*) and the total infected population $I(t)=\int_{\Omega }i(r,t)\;dr$. It is easy to see that, apart from a short initial transition period, the susceptible population is monotonically decreasing. This outcome can be expected, as for the fitness landscape that we used the incidence rate *k*(*r*) monotonically grows. The dynamics of the total infected population is more intriguing. Here, after a short transition period, there is an increase of the infected population due to increase of the incidence rate *k*. However, after reaching a certain level of *r*, the infected population (and hence the free phage population) is steadily decreasing. This decrease can be explained by the shortage of the resource (that is, by low abundance of the uninfected bacteria in this particular case).

It is noteworthy that the speed of the travelling wave in figure 1, and hence the speed of evolution, is not constant. It can be seen in figure 1 that initially evolution accelerates as the fitness grows. Then, when a certain level of the Darwinian fitness is reached, the evolution abruptly slow down. (In figure 1 this slow-down occurs at *t*≈500 days.) However, it can be seen that speed of evolution is not constant after this moment either. This outcome qualitatively matches previous results [7,10], where similar variations of the speed of evolution were observed for the HIV within-host evolution models. In contrast, in simulations with the TLK model the speed remains constant after an initial transition period.

It can be conjectured that the speed grows with the Darwinian fitness and with population size. The deceleration of evolution seen in figure 1 is likely caused by the reduction of the infected population and by the lack of available resources (that is, the susceptible bacteria), which is seen in figure 3*b*.

**Figure 4.**
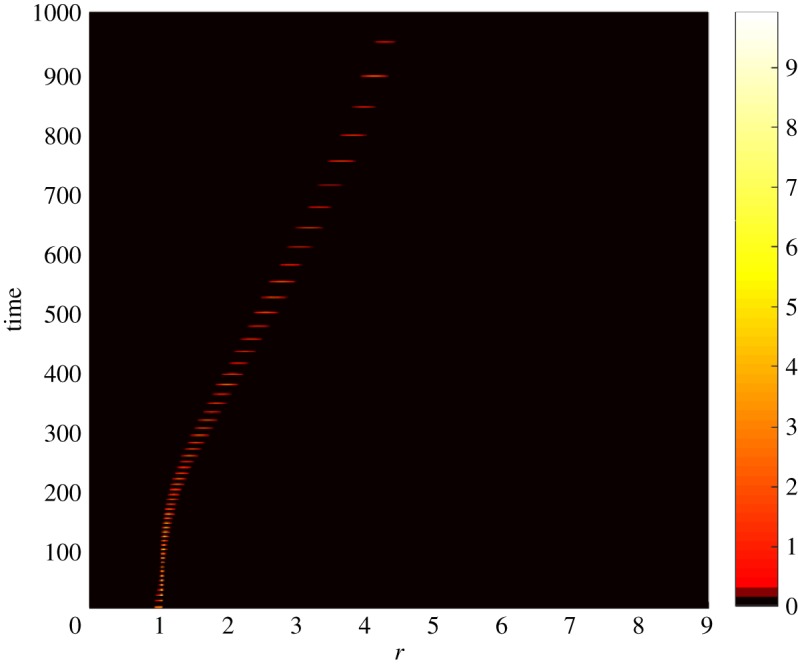
Density distribution of the infected population *i*(*r*,*t*) by viral phenotype in time. The colours correspond to the density magnitude; see the legend on the right-hand side. Please note the formation of a pulse-type travelling wave moving in the phenotype space in the right-hand direction. Here *q*=10^−6^ days^−1^, *α*=1.5 days^−1^, *C*= 100 cells ml^−1^, *λ*=3 days^−1^, *μ*=20 days^−1^, *b*=149.254 and *ξ*=0.02 ml cell day^−1^.

**Figure 5.**
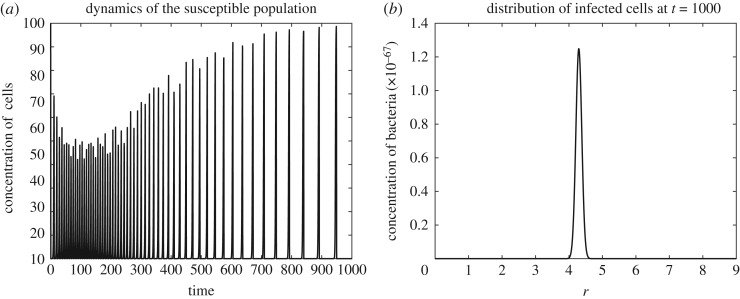
The dynamics of the susceptible bacterial population *S*(*t*) (*a*) and the density distribution of the infected population by viral phenotype *i*(*r*,*t*) at *t*=1000 days (*b*) for $R_{0}>R_{0}^{\mathrm{cr}}$.

As we mentioned above, the Beretta–Kuang model exhibits a supercritical Hopf bifurcation. That is, there is a critical value of the basic reproduction number $R_{0}^{\mathrm{cr}}$ such that a supercritical Hopf bifurcation occurs at $R_{0}=R_{0}^{\mathrm{cr}}$, and a stable limit cycle exists in the model phase space for all $R_{0}>R_{0}^{\mathrm{cr}}$. Figures 1–3 correspond to $R_{0}<R_{0}^{\mathrm{cr}}$. However, model (2.2) preserves this property of the original Beretta–Kuang model: that is, at $R_{0}=R_{0}^{\mathrm{cr}}$ model (2.2) loses its stability, and, as *R*_0_ grows further, self-sustained oscillations arise. Figures 4 and 5 show the dynamics of the system for this case. In these figures, *ζ*=0.02 ml cell day^−1^, while all other parameters are the same as in figures 1–3. Figure 4 shows the pulse-type travelling wave in the virus variant space: while self-sustained oscillations of the viral abundance are clearly seen in this figure, the wave propagation remains steady. The dynamics of the susceptible population for model (2.2) and a typical distribution of the infected population in the phenotype space *i*(*r*,*t*) are shown in figure 5 (*a* and *b*, respectively).

The simulations demonstrate that the self-sustained oscillations do not affect propagation of the travelling wave and, hence, the progress of viral evolution. It may appear initially that figures 1, 2, 4 and 5 show a slower evolution in the oscillating system. However, we have to remember that figures 1 and 2, on one side, and figures 4 and 5, on the other side, are produced for the fitness landscapes of different slopes. Specifically, figures 1 and 2 depict data for *ζ*=0.002 ml cell day^−1^, whereas figures 4 and 5 are for *ζ*=0.02 ml cell day^−1^. That is, in figures 1 and 2, for 1000 days the mean value of *k*(*r*)=*ζr* of the viral quasi-species changes from 0.002 to 0.0116 ml cell day^−1^ (and hence the mean value of *R*_0_ changes from 1.48 to 8.288), whereas in figures 4 and 5 for the same 1000 days the mean *k*(*r*) changes from 0.02 to 0.084 ml cell day^−1^ (and hence the mean *R*_0_ changes from 14.8 to 62.16). That is, in fact, changes of viral fitness in figures 4 and 5 occur faster. However, this acceleration of evolution most likely should be attributed to a higher fitness and to a considerably larger slope of the fitness landscape.

Please note that as the basic reproduction number *R*_0_(*r*) grows due to evolution, periods of the self-sustained oscillations grow as well. (This can be expected, as the basic reproduction number *R*_0_ is the bifurcation parameter for the Beretta–Kuang model (2.1), and the size of the limit cycle in the model phase space, as well as the period, grows as the bifurcation parameter grows.) Moreover, as the cycle in the phase space of ODE model (2.1) grows, it is squeezed to the coordinate planes. This implies that throughout a period, the infection levels remain very low most of the time. This accounts for the very low magnitude of distribution *i*(*r*,*t*) seen in figure 5 and potentially can lead to extinction of the virus or of both species.

## 4. Conclusion

The aim of this paper was to construct a reasonably simple model of viral evolution and to explore how particularities of a virus dynamics model, and in particular a host cell replication mode, affect viral evolution. To address this question, in this paper we constructed a model of aquatic bacteriophage evolution. This model is based on the Beretta–Kuang model of bacteriophage dynamics and is mechanistic (based on the first principles), at least as far as the Beretta–Kuang model is mechanistic. In contrast to the original Beretta–Kuang model, the model in this paper postulates existence of a multitude of viral variants with continuously distributed phenotypes that are arranged in a continuous phenotype space. Random mutations are modelled by the dispersion in the phenotype space.

The model constructed in this paper does not include any explicit law, or a mechanism, of evolution. The authors expect instead that, according to Darwinian principles, random mutations combined with natural selection and an uneven fitness landscape are sufficient to initiate viral evolution. One of authors’ intentions was to compare this model’s dynamics with that of the earlier considered model of within-host HIV evolution [7,9,11]. The latter model uses the same set of assumptions but is based upon the Nowak–May within-host HIV model. The principal difference between these two models of viral evolution is the target cell replication mode. While this difference may appear insignificant, it leads to different dynamics, as the paradox of enrichment is possible in the Beretta–Kuang model.

Numerical simulations with the bacteriophage evolution model exhibit the formation of a pulse-type travelling wave moving in the phenotype space towards the increasing Darwinian fitness (increasing *R*_0_). This confirms the expectation that on an uneven fitness landscape random mutations and natural selection (which in this model is provided by the conspecific competition for a limited resource) lead to the Darwinian evolution. It is noteworthy that the speed of this wave (and hence the speed of evolution) is not constant, but depends on the current viral fitness and the abundance of susceptible cells. These results qualitatively coincide with results obtained previously [7,9,11] for HIV within-host evolution, where travelling waves of evolution of varying speed were also observed.

The numerical simulations also exhibit the loss of stability and the rise of self-sustained oscillations in the bacteriophage evolution model (2.2), which correspond to the paradox of enrichment in the original Beretta–Kuang model and occur when *R*_0_ exceeds a certain critical value $R_{0}^{\mathrm{cr}}$. The authors have to note, however, that, while for *R*_0_ in the vicinity of $R_{0}^{\mathrm{cr}}$ the dynamics of model (2.2) closely resembles a supercritical Hopf bifurcation, and corresponds to that in the Beretta–Kuang model (2.1), the term ‘Hopf bifurcation’ in its precise meaning is not applicable to this case, as this model is not in an equilibrium state. Furthermore, the numerical simulations show that the only effect that the loss of stability and the following rise of the self-sustained oscillations have on the viral evolution is a deceleration of the evolution. No other effects, and in particular qualitative effects, were observed. This observation is the most important outcome of this study.

We also would like to stress that the simplistic fitness landscape that was used in this paper is for illustration purpose only and is not related to any real-life system. This simplest case, however, demonstrates that the Darwinian fitness of virus should monotonically increase on any uneven parts of more realistic fitness landscapes.

In this model, we completely disregard the possibility of co-evolution of the bacteria and phages and completely neglect bacterial evolution. Apart from our intention to keep the model as simple as possible, the major reason for this assumption is a considerable (of orders of magnitude) difference in the characteristic time scales of all processes (in particular reproduction and mutation rates) in the interacting viral and bacterial populations. We have to note, however, that the co-evolution can be considered in the same modelling framework: in order to do this, one has to assume existence of a multitude of bacterial phenotypes (continuously distributed, for the sake of consistency), define a model for the bacterial mutation mechanism (in the same way as it was done for the virus), define a bacterial phenotype space, *Ω*_*b*_, define a bacteria fitness landscape over this space, modify the viral fitness landscape postulating its dependence on bacterial phenotype, and then consider the same process in *Ω*×*Ω*_*b*_. While such a task is not impossible, it would require very large computational power. Nevertheless, despite these difficulties and because of these difficulties, modelling the co-evolution remains one of the most exciting problems in evolutionary biology [21–24].

## Data accessibility

The code can be found at https://doi.org/10.5061/dryad.hk64f [25].
